# Experimental hookworm infection and escalating gluten challenges are associated with increased microbial richness in celiac subjects

**DOI:** 10.1038/srep13797

**Published:** 2015-09-18

**Authors:** Paul Giacomin, Martha Zakrzewski, John Croese, Xiaopei Su, Javier Sotillo, Leisa McCann, Severine Navarro, Makedonka Mitreva, Lutz Krause, Alex Loukas, Cinzia Cantacessi

**Affiliations:** 1 Centre for Biodiscovery and Molecular Development of Therapeutics, Australian Institute of Tropical Health and Medicine, James Cook University, Cairns, QLD, Australia; 2 Bioinformatics Laboratory, QIMR Berghofer Medical Research Institute, Brisbane, QLD, Australia; 3 Prince Charles Hospital, Brisbane, QLD, Australia; 4 Department of Veterinary Medicine, University of Cambridge, Cambridge, United Kingdom; 5 The Genome Institute, and; 6 Department of Medicine, Washington University School of Medicine, St. Louis, MO, USA; 7 University of Queensland Diamantina Institute, Translational Research Institute, Woolloongabba, QLD, Australia

## Abstract

The intestinal microbiota plays a critical role in the development of the immune system. Recent investigations have highlighted the potential of helminth therapy for treating a range of inflammatory disorders, including celiac disease (CeD); however, the mechanisms by which helminths modulate the immune response of the human host and ameliorate CeD pathology are unknown. In this study, we investigated the potential role of alterations in the human gut microbiota in helminth-mediated suppression of an inflammatory disease. We assessed the qualitative and quantitative changes in the microbiota of human volunteers with CeD prior to and following infection with human hookworms, and following challenge with escalating doses of dietary gluten. Experimental hookworm infection of the trial subjects resulted in maintenance of the composition of the intestinal flora, even after a moderate gluten challenge. Notably, we observed a significant increase in microbial species richness over the course of the trial, which could represent a potential mechanism by which hookworms can regulate gluten-induced inflammation and maintain intestinal immune homeostasis.

The human gut is inhabited by ~1000 species of bacteria (i.e. the gut microbiota) that play essential roles in human health, including nutrient metabolism, protection against pathogens and regulation of both innate and adaptive immune responses^1^,^2^,^3^. The nature and composition of the intestinal microbiota is subject to a range of factors, including host genetics, immune status, diet and co-infections with extraneous pathogens^4^. Perturbations in the symbiotic relationship that exists between the commensal microbiota and the host immune system have been associated with a number of chronic inflammatory and metabolic diseases of the gastrointestinal tract, including obesity, diabetes, inflammatory bowel disease (IBD) and celiac disease (CeD), disorders that are becoming increasingly prevalent, particularly in the developed world^5^,^6^,^7^. For example, both the concentrations of mucosally-associated bacteria and the nature of the bacterial species are distinct in individuals with IBD, with substantial depletions in Bacteroidetes and Lachnospiraceae and expansion of populations of Actinobacteria and Proteobacteria^8^,^9^. Similarly, CeD (an autoimmune disorder characterised by an inappropriate immune response to dietary gluten) is associated with increased populations of *Bifidobacterium* spp. and *Staphylococcus* spp. and reductions in *Bacteroides* spp.^10^. Hence, addressing the microbiological alterations in patients with these chronic inflammatory disorders may be a viable therapeutic strategy, with some encouraging findings from a limited number of small studies^11^,^12^,^13^.

One of the hypotheses as to why allergic and autoimmune diseases of the gastrointestinal tract, such as food allergy and CeD, are increasing is the change in relative exposure to commensal and pathogenic organisms as a consequence of urbanization and improved sanitation in the developed world^14^. It has been proposed that re-introduction of some of these infectious organisms, and particularly of parasitic helminths, could lead to novel therapies to treat autoimmune diseases^15^,^16^,^17^,^18^,^19^,^20^,^21^,^22^. One of the mechanisms by which helminths have been proposed to regulate immune homeostasis is via modulation of bacterial communities in the intestine^23^,^24^,^25^. However, these studies were performed in animal models of helminth infection and it is unclear if similar mechanisms operate in humans.

We have recently demonstrated that experimental infection with the human hookworm, *Necator americanus*, improved gluten tolerance in CeD subjects^22^. *N. americanus* infections were associated with suppression of pro-inflammatory cytokines and increase of both the frequency of immune-regulatory cells and cytokines in the gut following gluten challenges^19^,^22^,^26^; however, the mechanism/s by which hookworms modulate the immune response of their human hosts, thus potentially ameliorating the symptoms of chronic inflammatory disorders of the intestinal tract, are yet to be fully understood. In particular, given the role played by gut dysbiosis in the pathogenesis of CeD^27^, it is possible that the ‘curative’ properties of hookworms are partly associated with the ability of these parasites to induce qualitative and/or quantitative modifications in the gut microbiota, thus contributing to the restoration of intestinal immune homeostasis. To address this hypothesis, we recently investigated the impact that experimental infections of healthy CeD subjects (on a gluten-free diet, GFD) with *N. americanus* exert on the composition of the gut microbiota and the relative abundance of different microbial species^28^. No detectable qualitative and quantitative differences were observed between samples prior to and 8 weeks following hookworm infection, thus indicating that the microbial community composition of each subject remained stable over the relatively short timeframe of the study^28^. However, the changes in gut microbiota of hookworm-infected CeD subjects following gluten challenge was not investigated. In the present study, capitalising on the sampling opportunities provided by our published clinical trial^22^, we explore the qualitative and quantitative changes in gut microbiota of CeD subjects prior to and following experimental infection with *N. americanus*, as well as following the administration of escalating doses of gluten.

## Results

### The fecal microbiota of Trial subjects differs from that of active CeD control subjects

**Figure 1:**
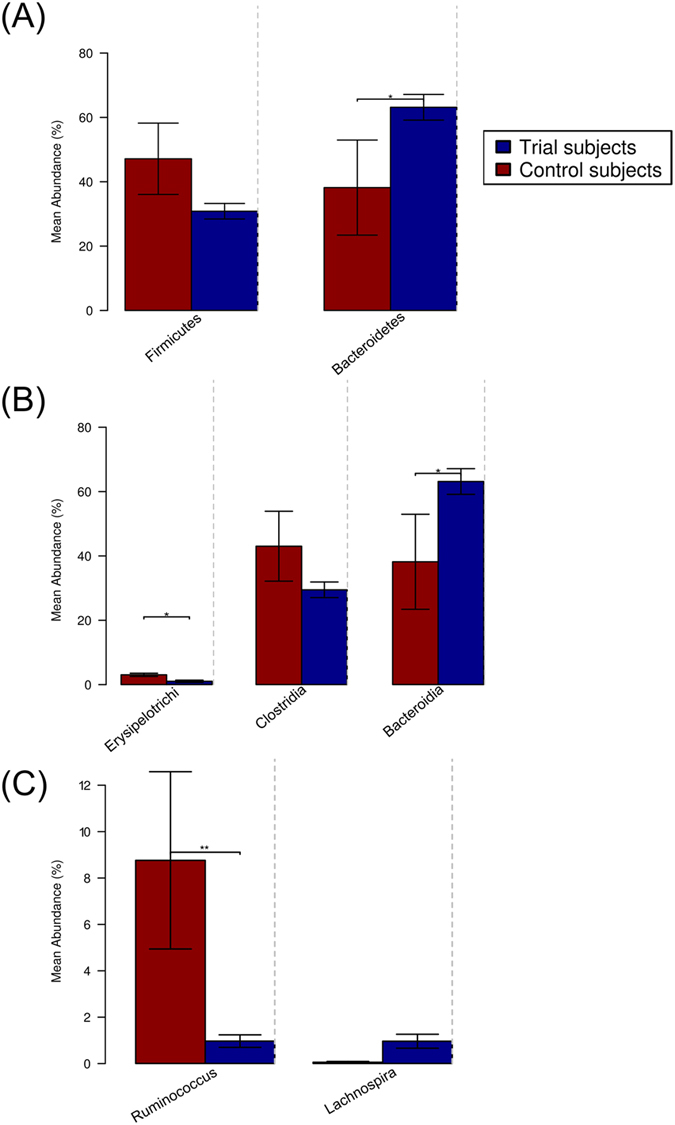
The fecal microbiota of Trial subjects differs from that of active CeD control subjects. Differences in the composition of the fecal microbiota of Trial subjects prior to hookworm infection (T0) and of active celiac disease Control subjects were determined at (**A**) phylum, (**B**) class and (**C**) genus level. *p < 0.05, **p < 0.01.

**Figure 2:**
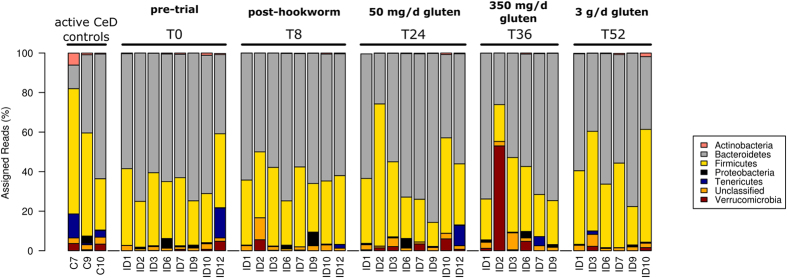
Composition of the fecal microbial communities (at phylum level). Microbial composition of fecal samples from Trial subjects (ID*) prior to and following infection by *Necator americanus* (T0 and T8, respectively), as well as following administration of escalating doses of gluten (50 mg/day – T24; 350 mg/day – T36; 3 g/day – T52) as predicted in the analysis of the V1-V3 16S rRNA gene. The composition of the microbial communities detected in fecal samples from Control subjects with active celiac disease (C*) is also shown.

Each of the eight Trial subjects had been on a strict GFD for at least 5 years prior to trial commencement, and recorded sub-clinical Marsh scores prior to entering the trial^22^. We first assessed the baseline pre-trial (T0) composition of the fecal microbiota of the Trial subjects and compared it with that of a cohort of individuals from the same metropolitan area with active Marsh 3 + grade CeD (Table S1). Trial subjects displayed a greater abundance of the phylum Bacteroidetes than Control subjects (t-test: p = 0.04; FDR = 0.12), while Firmicutes were more abundant in the Control subjects (p = 0.07; FDR = 0.12, Figs 1A and 2). In addition, Tenericutes RF39 were detected in the fecal microbiota of all Control subjects, but only in one Trial subject (ID12; Fig. 2). At the class level, Bacteroidia were more prevalent in the Trial subjects (p = 0.04; FDR = 0.17), whereas Erysipelotrichi were more abundant in the Control subjects (p = 0.02; FDR = 0.17), as were Clostridia, although the differences did not reach significance (p = 0.13; FDR = 0.2, Fig. 1B). Similarly, at genus level, *Ruminococcus* dominated the fecal microbiota of the Control subjects (p = 0.001; FDR = 0.02, Fig. 1C), while *Lachnospira* was more abundant in the Trial subjects (p = 0.027; FDR = 0.16) (Fig. 1C). Comparison of Kyoto Encyclopedia of Genes and Genomes (KEGG) functional profiles (predicted by PICRUSt) of Trial subjects pre-trial with those of the Control subjects displayed differential regulation of 8 biological pathways, with up-regulation of pathways linked to sugar uptake (i.e. “Multiple sugar transport system permease protein”, KO codes K02026 and K02025; p < 0.03; FDR = 0.2) in the Control subjects (Figure S1), associated with bacteria within the genus *Ruminococcus* (data not shown). Together, these data demonstrated that the microbiota of the healthy Trial subjects pre-hookworm and pre-gluten challenge was distinct from individuals with active CeD.

### Changes in fecal microbiota following hookworm infection and gluten challenge revealed by targeted gene sequencing

**Figure 3:**
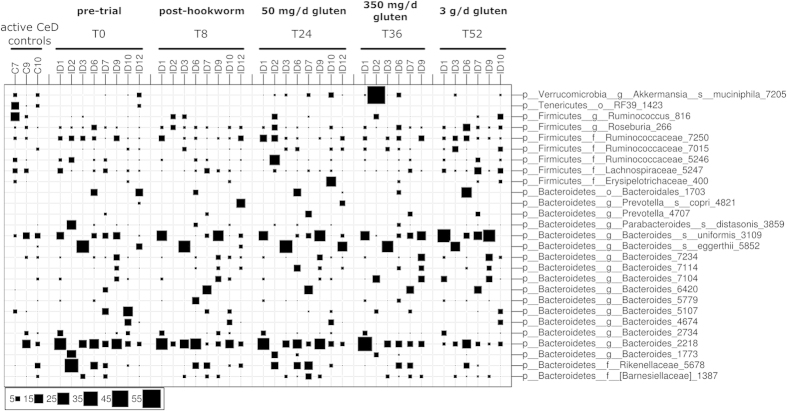
Composition of the fecal microbial communities at phylotype level. Microbial composition of fecal samples from Trial subjects (ID*) prior to and following infection by *Necator americanus* (T0 and T8, respectively), as well as following administration of escalating doses of gluten (50 mg/day – T24; 350 mg/day – T36; 3 g/day – T52) as predicted in the analysis of the V1-V3 16S rRNA gene. Bubble sizes reveal the relative abundance (%) of phylotypes (based on 97% sequence identity) in each sample. The composition of the microbial communities detected in fecal samples from Control subjects with active celiac disease (C*) is also shown. OTUs listed were present in at least one sample at a relative abundance of ≥7%.

We next aimed to assess whether the nature of the commensal makeup was altered in the Trial subjects following experimental hookworm infection and subsequent escalating gluten challenges. All eight subjects were successfully infected with *N. americanus*, as evidenced by positive detection of hookworm eggs in the feces from weeks 8 to 52 post-infection^22^. Fecal samples were collected from each of these subjects 8 weeks after hookworm infection (T8), as well as at defined intervals following sequential challenges with 50 mg gluten/day for 12 weeks (T24), 350 mg gluten/day for 12 weeks (T36) and 3 g gluten/day for 2 weeks (T52). All samples were subjected to 454-based metagenomic sequencing; however, two Trial subjects did not provide fecal samples at either T52 (subject ID2) or T36 and T52 (ID12), hence sequence data was not available for these individuals at these time points. In addition, as the raw sequence data for Trial subject ID10 at T36 consisted of a total of 18 sequences only, this sample was excluded from subsequent analyses. A total of 346,669 useable reads were assigned to 7,262 Operational Taxonomic Units (OTUs) and 6 bacterial phyla, respectively (data available from the corresponding author upon request). Consistent with previous investigations, including our own^28^, the phyla Bacteroidetes and Firmicutes dominated the intestinal microbiota of all subjects included in the study at all time points investigated (Figs 2 and 3), with substantial inter-individual differences observed at all time points with regard to some of the less abundant phyla such as Proteobacteria, Tenericutes and Verrucomicrobia (Fig. 2). Further analysis of specific OTUs within the major phyla of Bacteroidetes and Firmicutes again revealed substantial inter-individual variation at all time points examined (Fig. 3).

### Hookworm infection and gluten challenge is associated with increased microbial richness

**Figure 4:**
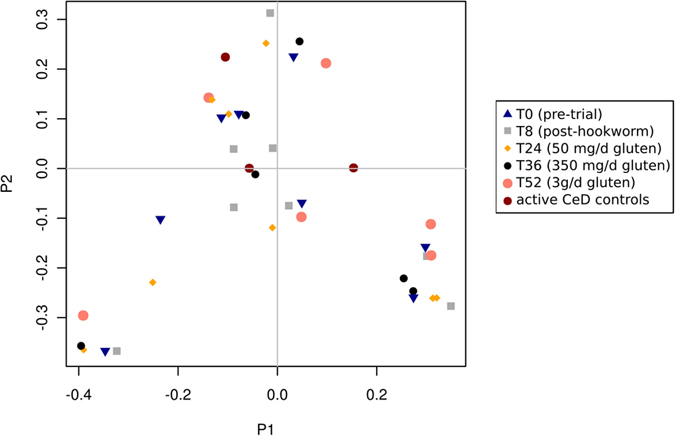
Principal coordinates analysis (PCoA) reveals stable composition of fecal microbial communities over time. The fecal microbiota of Trial subjects prior to and following experimental infection with *Necator americanus*, and following administration of escalating doses of gluten was assessed by PCoA. The composition of the microbial communities detected in fecal samples from Control subjects with active celiac disease is also shown. Community similarity was calculated using the Jaccard distance measure of the phylotypes.

**Figure 5:**
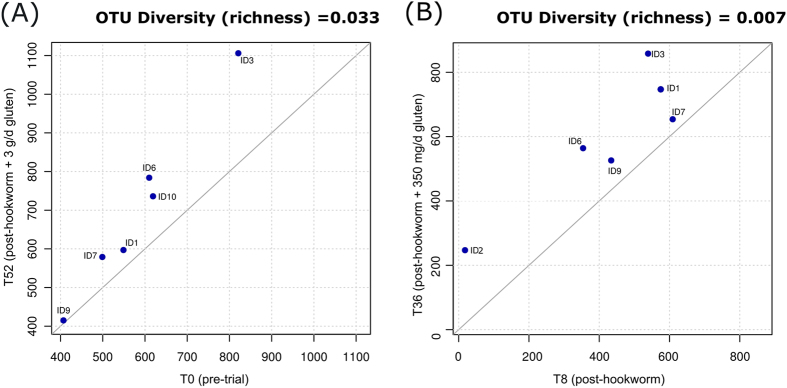
Hookworm infection and gluten challenge is associated with increased microbial richness. Pairwise comparisons depicting the overall taxonomic richness of the fecal microbiota of Trial subjects (**A**) prior to hookworm infection (T0) and following the administration of escalating doses of gluten (T52), and (**B**) post-hookworm infection (T8) and post-gluten challenge (T52), respectively.

Consistent with the results of our previous study^28^, a Principal Coordinates Analysis (PCoA) of the fecal microbial communities detected in the Trial subjects showed strong clustering of the samples by individual rather than infection status and/or exposure to escalating doses of gluten in the two main dimensions (Fig. 4), thus indicating that the community composition of each subject remained stable over the course of the trial. Similarly, both Anosim and Redundancy Analysis (RDA) indicated a clear clustering of samples by individual (p = 0.001 Anosim; p < 0.001 RDA). In the Trial subjects, hookworm infection and/or exposure to escalating doses of gluten did not impact community structure (p > 0.1 Anosim, p > 0.16 RDA), which is indicative of an inter-individual variability and intra-individual stability of the gut microbiota despite hookworm infection and gluten challenge. In line with this observation, there was no significant difference in relative abundance of any individual OTU identified in the study (97% sequence identity) across time points (FDR > 0.54 with both t-test and Wilcoxon test). While no significant difference in Shannon diversity was observed on the global microbial communities over the whole course of the study (p = 0.91, ANOVA), we detected a significant increase in bacterial species richness at T52 (following hookworm infection and exposure to 3 g/day of gluten) when compared to T0 (prior to hookworm infection) (p = 0.033, paired t-test) and at T36 (following hookworm infection and exposure to 350 mg/day gluten) when compared to T8 (following hookworm infection but prior to gluten challenge) (Fig. 5) (p = 0.007). Together, these data suggest that hookworm infection supports maintenance of the composition of the intestinal flora in the presence of escalating gluten challenges, with concomitant significant increases in microbial species richness.

## Discussion

In our recent study^22^, we showed that experimental infections with *N. americanus* enabled individuals with CeD to tolerate escalating challenges with dietary gluten. The biological, immunological and molecular mechanisms underlying this outcome are yet to be fully determined; however, we hypothesized that an expansion of regulatory T cells, together with reductions in pro-inflammatory effector T cells in the duodenum, may have been responsible for the restoration of the intestinal homeostasis and, consequently, for the improved gluten tolerance^19^,^22^,^26^. However, other elements within the complex spectrum of host-parasite interactions may have contributed to this cascade of biological events in the study subjects. Here, we evaluated the impact of hookworm infection and gradual re-introduction of gluten into the diet on the microbial communities inhabiting the intestine of the same cohort of volunteers who took part in the clinical study^22^.

We had hypothesized that experimental hookworm infection, combined with exposure to escalating doses of gluten, would result in significant quantitative and qualitative fluctuations of the gut microbiota populations of the volunteers. However, we detected no significant differences in the overall community structure, Shannon diversity or relative abundance of individual bacterial species over the course of our study, in accordance with the findings of our previous investigation in which a 8-week, acute hookworm infection alone did not result in significant alterations of the fecal microbiota^28^. However, in the present study, we observed a significantly increased microbial richness (= total number of microbial species present) in fecal samples of Trial subjects collected following hookworm infection and moderate gluten challenge, compared with pre-trial samples. One possible explanation for this discrepancy is that hookworms may actively contribute to the maintenance of bacterial homeostasis in the gut^28^ and ‘restore’ homeostasis (by promoting an increase in microbial richness) when inflammatory responses are triggered (e.g. by the introduction of gluten in a celiac subject). Alternatively, it is possible that hookworm-induced changes in microbial species richness can only be detected following the establishment of chronic, rather than acute, infections^28^. The latter theory is also substantiated by the results of a recent investigation, in which a significantly increased species richness was detected in the intestinal microbiota of individuals from indigenous Malaysian communities naturally infected with gastrointestinal helminths (*Trichuris* and/or hookworms and/or *Ascaris* sp.) when compared with helminth-free, uninfected subjects from the same areas^29^. Similarly, a study using a primate model of chronic idiopathic diarrhea showed that experimental infections with whipworms were associated with a significant increase of microbial species richness and evenness (= diversity) which, in turn, had been hypothesized to play a role in the restoration of intestinal homeostasis and symptomatic improvement^24^. In our study, the increase in species richness that followed hookworm infection was not associated with an increase in Shannon diversity (p > 0.05). This finding may be the result of limited statistical power as direct consequence of our small sample size, which may also have been responsible for the small (albeit significant) differences in microbial species richness observed over the course of the study. Nevertheless, a higher species richness of the gut microbiota has generally been associated with a “healthier” intestinal homeostasis^30^,^31^,^32^. In particular, a study comparing the intestinal microbiota of subjects suffering from Crohn’s Disease and Ulcerative Colitis with that of healthy controls revealed that species richness was significantly higher in the latter^30^. In addition, the microbiota isolated from non-inflamed tissue from diseased subjects displayed significantly increased species richness when compared with that from inflamed biopsy samples from the same individuals^30^. Therefore, based on the results of the present and previous studies that associate increased microbial species richness to infections with helminths^29^ and the amelioration of inflammation^24^,^30^,^31^,^32^, we hypothesize that the therapeutic properties of hookworms may be partly linked to their ability to promote species richness and restore/maintain microbial (and immune) homeostasis in the gastrointestinal tract^28^. However, one limitation of our clinical study^22^ was the unavailability of a hookworm- or gluten-placebo cohort; therefore, larger placebo-controlled trials, together with the definition of the enterotype of each subject^33^, are necessary to confirm whether hookworms alone, or the gradual reintroduction of dietary gluten, are involved in modulation of the microbiota.

Three fecal samples from volunteers with active CeD (Control) were included in our study and compared with the pre-trial (T0) samples from the Trial subjects. Quantitative differences in the abundance of the main phyla were observed between the two groups; in particular, the Firmicutes dominated the microbiota of the Control subjects with active CeD (albeit this difference was below statistical significance), whereas the Bacteroidetes were more abundant in the healthy Trial subjects. Despite this overall trend, the genus *Lachnospira* (a Firmicutes) was more abundant in the Trial subjects (but below statistical significance); Clostridia and *Ruminococcus* (both within the phylum Firmicutes) dominated the fecal microbiota of the Control subjects. A shift in the Bacteroidetes to Firmicutes ratio in association with active CeD has already been documented in a previous study examining the effects of sustained GFD on the composition of the duodenal microbiota of adult CeD volunteers^6^,^13^. Given the recently described roles of Firmicutes in the metabolism of gluten^34^, the abundance of bacteria within this phylum in subjects with active CeD may reflect the dietary habits of these volunteers. In contrast, the abundance of Clostridia and *Ruminococcus* (at the class and genus level, respectively), in the Control subjects is in disagreement with previous findings reporting *Methylobacterium* (class Alphaproteobacteria) and *Mycobacterium* (Actinobacteria) as the prevalent genera of bacteria in the gut microbiota of adults with active CeD^6^. One possible explanation for this discrepancy is technical, and likely to be associated to methodological differences between our study and that by Nistal *et al.*^6^; indeed, the latter utilized PCR amplification of the bacterial 16 S rRNA gene, followed by cloning and sequencing of randomly-picked clones, yielding ~50 sequences per sample. Therefore, in our opinion, the vast difference in sequencing depth between our study and that by Nistal *et al.*^6^ does not warrant direct comparisons of findings. However, a higher prevalence of Clostridia had been detected in the feces of CeD children with active disease compared with CeD controls on a strict GFD^35^. In addition, a recent investigation of the fecal microbiota of infants at either high- or low- genetic risk of developing CeD (HLA-DQ2 carriers and non-HLA-DQ2 carriers, respectively) revealed that the former is characterized by significantly higher proportions of Clostridia^27^. However, the role of this bacterial group in the progression of CeD is yet to be ascertained.

In conclusion, the present study has revealed a potential beneficial consequence of hookworm infection in the context of an autoimmune disease, i.e. supporting the maintenance and diversity of commensal flora. Whether hookworm-mediated changes in the microbiota are causal for the improved gluten tolerance and reduced inflammation in CeD is unknown, but could be examined in greater detail in an animal model of the disease. It also remains possible that hookworm-induced immunoregulation is mediated by additional mechanisms, in particular the direct actions of parasite excretory/secretory proteins on immune cells^36^. Clearly, helminth-based therapies hold promise for a range of autoimmune or inflammatory conditions, and reversal of the dysbiosis associated with these disorders by tissue-resident parasites is one potential mechanism that requires further investigation.

## Methods

### Ethics statement

This study was approved and carried out in strict accordance and compliance with the National Statement on Ethical Conduct in Research Involving Humans guidelines of the National Health and Medical Research Council of Australia (NHMRC). The Prince Charles Hospital (Brisbane, Australia) and James Cook University Human Research Ethics Committees approved the study. Written informed consent was obtained from all subjects enrolled in the study. This study was registered as a clinical trial at ClinicalTrials.gov as NCT00671138^22^.

### Trial design

Eight otherwise healthy volunteers with HLA-DQ2 + or HLA-DQ8 + CeD on a strict GFD (>5 years) (= Trial subjects) were infected percutaneously with 20 infective third stage larvae of *N. americanus*^26^. Subjects then underwent escalating exposure to dietary gluten (as spaghetti), with a 10–50 mg/day micro-challenge from weeks 12–24, followed by intermittent twice weekly 1 g/day gluten challenge from weeks 24–36 and a 3 g/day challenge from weeks 48–50^22^. Prior to experimental infection (T0), as well as at 8 weeks (T8), 24 weeks (T24), 36 weeks (T36) and 52 weeks (T52) post-infection, individual fecal samples were collected from each subject and stored at –20 °C. In addition, individual fecal samples from three hookworm-naïve volunteers with active CeD (diagnosed as Marsh grade 3 at the time of biopsy^22^) (= Control subjects) were also included for comparative purposes (Table S1).

### DNA extraction and 16S rRNA pyrosequencing

Genomic DNA was extracted directly from each sample using the PowerSoil^TM^ DNA isolation kit (MoBio, USA), according to the manufacturer’s instructions. The V1-V3 hypervariable region of the 16S gene was PCR amplified using universal primers described previously^28^ and sequenced on a 454 GS-FLX Titanium platform (Roche). Forward primers incorporated GS Titanium adapters as well as a sample-specific barcode sequences. Raw sequence data have been deposited in the NCBI Sequence Read Archive under accession number SRP059769.

### Bioinformatics analyses

Sequence data were processed using the Quantitative Insights Into Microbial Ecology (QIIME) software suite^37^. Briefly, after filtering of low-quality reads, all remaining sequences were de-multiplexed, and chimeric sequences were removed using UCHIME v 3.0.617. Sequences were subsequently clustered into Operational Taxonomic Units (OTUs) on the basis of similarity to known bacterial sequences in the Greengenes database (v13.5; http://greengenes.secondgenome.com/; cut-off: 97% sequence similarity) using the UCLUST software; then, sequences were assigned to taxonomy using the Ribosomal Database Project (RDP) Classifier with the confidence level set at 0.8. Statistical analyses and data mining were conducted using the Calypso software (http://bioinfo.qimr.edu.au/calypso/). Shannon diversity and rarefied richness (rarefy function in vegan Bioconductor package) were compared by paired t-test and rarefaction curves. Differences in abundance of individual OTUs were assessed by paired t-test on square root transformed relative abundance values and Wilcoxon test (Table S2), and p-values were corrected for multiple testing by false discovery rate (FDR). Anosim (Jaccard distance), redundancy analysis (RDA; including human subject and week as explanatory variables), Principal Coordinates Analysis (PCoA; Jaccard distance) and rarefaction analyses were run in Calypso with default parameters. Anosim and RDA were applied on the OTU table using Jaccard distance. RDA was executed including the environment variables infection status and subject ID. Anosim was run separately for infection status and subject ID. The software PICRUSt^38^ was then used to predict metagenomic functional content from the Greengenes-picked OTUs and predict KEGG pathway abundance. Briefly, sequences were clustered into OTUs using the reference database Greengenes (v13.5). Reference OTUs were normalized by copy number, and PICRUSt metagenome predictions were calculated.

## Additional Information

**How to cite this article**: Giacomin, P. *et al.* Experimental hookworm infection and escalating gluten challenges are associated with increased microbial richness in celiac subjects. *Sci. Rep.* **5**, 13797; doi: 10.1038/srep13797 (2015).

## Supplementary Material

Supplementary Information
