# Supplementary material for: Experimental hookworm infection and escalating gluten challenges are associated with increased microbial richness in celiac subjects
Source: Sci Rep. 2015 Sep 18;5:13797. doi: 10.1038/srep13797 (PMC4585380; doi:10.1038/srep13797)
Supplement: Supplementary Information [file srep13797-s1.pdf]

Supplementary information

Giacomin, P., Zakrzewski, M., Croese, J., Su, X., Sotillo J., McCann, L., Navarro, S., Mitreva, M., Krause, L., Loukas, A. & Cantacessi, C. Experimental hookworm infection and escalating gluten challenges are associated with increased microbial richness in celiac subjects.

Figure S1

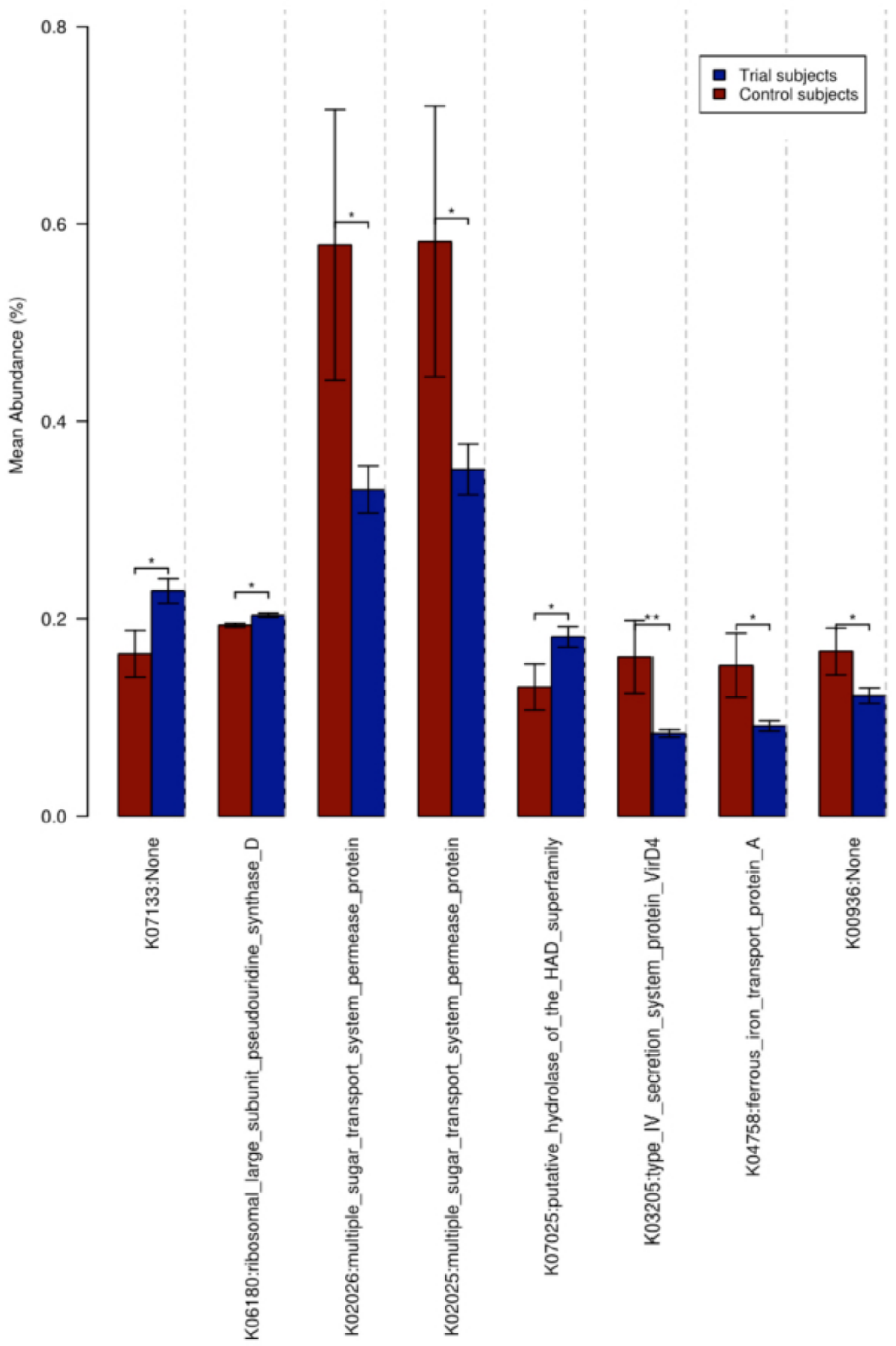

Figure S1: Differences in PICRUSt predicted (KEGG) metabolic pathways inferred from the fecal microbial communities of Trial subjects prior to hookworm infection (T0) and of active celiac disease Control subjects.

Table S1

**Subject**	**Age**	**Sex**	**Marsh score**
ID1	43	F	M0
ID10	51	M	M2
ID12	67	F	M0
ID2	39	F	M0
ID3	47	F	M0
ID6	39	M	M0
ID7	63	M	M0
ID9	62	F	M1
C7	53	F	M3b
C9	29	F	M3a
C10	76	M	M3b

Table S1: Table of trial subject identification (ID) numbers or control subject (C) numbers displaying age, sex and baseline clinical diagnosis (T0 Marsh Score).

Table S2

rank	taxon	t-test p-value (FDR)*	Rank test p-value (FDR)**	Median Control	Median Trial
phylum	Bacteroidetes	0.04 (0.13)	0.13 (0.22)	39.55	63.48
	Firmicutes	0.07 (0.13)	0.19 (0.22)	52.14	31.62
Class	Bacteroidia	0.04 (0.17)	0.13 (0.26)	39.55	63.48
	Erysipelotrichi	0.02 (0.17)	0.048 (0.2)	3.5	0.64
	Clostridia	0.13 (0.23)	0.28 (0.37)	48.63	30.88
Genus	Ruminococcus	0.001 (0.03)	0.01 (0.13)	6	0.8
	Lachnospira	0.03 (0.25)	0.01 (0.13)	0.04	0.7

*based on square root transformed relative taxa counts
**based on relative taxa counts

Table S2: Comparison of taxa at phylum, class and genus level between Control and Trial subjects.
